# A simulation environment for studying transcutaneous electrotactile stimulation

**DOI:** 10.1371/journal.pone.0212479

**Published:** 2019-02-22

**Authors:** Gloria Araiza Illan, Heiko Stüber, Ken E. Friedl, Ian R. Summers, Angelika Peer

**Affiliations:** 1 EPSRC Centre for Doctoral Training in Future Autonomous and Robotic Systems (FARSCOPE), Bristol Robotics Laboratory, University of Bristol, Bristol, United Kingdom; 2 Bristol Robotics Laboratory, University of the West of England, Bristol, United Kingdom; 3 Chair of Information-oriented Control, Department of Electrical and Computer Engineering, Technical University of Munich, Munich, Germany; 4 Chair of Automatic Control Engineering, Department of Electrical and Computer Engineering, Technical University of Munich, Munich, Germany; 5 Faculty of Science and Technology, Free University of Bozen - Bolzano, Bruneck - Brunico, Italy; Universidad de Salamanca, SPAIN

## Abstract

Transcutaneous electrical nerve stimulation (TENS) allows the artificial excitation of nerve fibres by applying electric-current pulses through electrodes on the skin’s surface. This work involves the development of a simulation environment that can be used for studying transcutaneous electrotactile stimulation and its dependence on electrode layout and excitation patterns. Using an eight-electrode array implementation, it is shown how nerves located at different depths and with different orientations respond to specific injected currents, allowing the replication of already reported experimental findings and the creation of new hypotheses about the tactile sensations associated with certain stimulation patterns. The simulation consists of a finite element model of a human finger used to calculate the distribution of the electric potential in the finger tissues neglecting capacitive effects, and a cable model to calculate the excitation/inhibition of action potentials in each nerve.

## Introduction

Mechanoreceptors transform mechanical stimuli into electrical signals. In the human body, they are distributed in the skin, muscles, joint capsules, viscera and tendons [1]. Tactile sensations (texture, pressure, vibration, etc.) result from the excitation of cutaneous somatosensory receptors, such as Merkel cells, Meissner, Pacinian and Ruffini corpuscles.

Practically all interactions with objects involve the excitation of a large number of sensory units [2, 3]. The mechanical stimulus produces a change in the electric membrane potential of both the receptor and the nerve fibre connected to it. If the membrane potential rises above the excitation threshold, an action potential (AP) is induced, which will then lead to the transmission of the signal towards the Central Nervous System (CNS).

Transcutaneous electrical nerve stimulation (TENS) is an established technique, used as a research tool in domains such as neuroscience [4]. TENS can produce tactile sensations, stimulating nerve fibres connected to the skin mechanoreceptors through electrodes on the skin, but has not yet found its way into consumer applications as the relationship between more complex stimulation patterns and achieved sensation is not fully understood yet. Devices based on TENS would give the opportunity to increase the amount of information that systems could supply for medical, teleoperation, industrial and gaming applications, i.e. providing haptic feedback. TENS can offer advantages over the alternative of mechanical stimulation systems [5, 6], which typically involve a complex hardware expensive to produce and maintain.

In order to investigate transcutaneous nerve stimulation and its dependence on electrode layout and excitation patterns, it is necessary to have a theoretical description of the electrical behaviour of the human skin, nerves and related tissues. Various models describing nerve excitation have been developed since the second half of the 19th century, showing the theoretical nerve response to a stimulus and its propagation through time and space. Commonly used nerve representations are the cable model [7] and the Hodgkin and Huxley model [8], which explain the electrical dynamics of nerve fibres through a set of differential equations. In addition, electrical properties of human skin and underlying tissues have been analysed and documented in diverse histological studies, providing further information that is required to successfully model a TENS system.

This paper introduces a simulation framework that can be used as a research tool to study TENS systems. Specifically, we demonstrate in two simulation-based scenarios its use for the mathematical modelling of observed experimental findings and the simulation-based formulation of new hypotheses, which can form the basis for new experimental studies. These hypotheses involve the selective stimulation of specific nerves located at different depths.

## Related work

TENS has been used to study the effects of defined stimulation patterns applied to specific nerves in different body parts. It has been implemented to produce tactile sensations on the fingers [9], tongue [10] and hands [11], and was applied to arm muscles to induce their contraction and relaxation [12]. It has also been employed for the treatment of pain [4, 13, 14] and in relation to the auditory system, to treat tinnitus or improve sound perception [15].

Research groups have also been studying and developing systems to excite nerves which carry information from mechanoreceptors in the skin. Kajimoto et al. [16, 17] developed a system with the intention of selectively stimulating three different types of mechanoreceptors. The nerve fibres were represented by a cable model and the predicted response was compared to users’ subjective perception of the various stimuli. They showed in their simulation that when deeper nerve fibres were targeted for stimulation, unwanted stimulation of shallower fibres was also produced.

Likewise, use of a finite element model (FEM) of the skin and underlying tissues in conjunction with nerve fibre models, has been an area of interest. Kuhn [12, 18, 19] modelled a TENS system for the human arm, using a FEM to study the effect on nerve selectivity from changing the electrodynamic properties of the skin (such as resistivity and permittivity) and the size of the electrode array. He implemented five different nerve-fibre models linked to the FEM: a non-linear cable model, a non-linear temperature-compensated cable model, a non-linear mammalian nerve fibre model, a non-linear double cable model and a linear double cable model. The results of each model were compared to the user’s muscular activation when the stimulus was presented to motor nerves, together with electrode measurements of intramuscular potential and potential on the skin surface. It was concluded that a non-linear cable model, where the nodes of Ranvier, paranodal and internodal sections were included, was the most realistic.

The simulation environment described in the present paper is a representation of the human finger giving a setup that can be used to study TENS, more specifically to systematically design and test new TENS devices and evaluate the effect of using different stimulation patterns. The results in this manuscript show that by suitable choice of the electrode currents (stimulation pattern), a specific nerve fibre can be selectively stimulated at different depths without exciting other fibres. Using this environment, it is possible to replicate previous reported experimental findings and to propose and discuss new hypotheses regarding tactile perception and their relation to different stimulation patterns.

## Materials and methods

Our environment consists of a finite element electrical model of the human finger connected to a representation of the nerve response, based on the cable model. It can be used to analyse the specific behaviour of a nerve fibre in response to a particular distribution of stimulation currents at the surface of the skin. Advantages of the FEM include the generation of the modelled human finger’s physical response at any location, taking account of local variations of electrical properties, which can sometimes be neglected by analytical approaches. The model also allows calculation of the time-varying activation of fibres in response to complex time-varying stimuli. Overall, it offers a rapid analysis of performance and evaluation of design parameters for virtual prototyping of TENS systems, by providing a visual representation and calculation of physical parameters simultaneously.

### 1 Electrical field model

A FEM of a human finger with a cylindrical geometry and a spherical fingertip is used to compute the current and electric field distributions generated by a TENS system. The FEM was developed using Elmer (https://www.csc.fi/web/elmer) and Gmsh (http://www.gmsh.info) software. The FEM is segmented into tetrahedral elements, each treated as a volume conductor with one of three values for conductivity *σ*_*bone*_, *σ*_*fat*_ or *σ*_*skin*_, as appropriate (the pulp of the finger is taken to be fat throughout; in fact, it is composed of fibrous septa filled with fat [20, 21]). The skin is set to be dry. The model considers three nerve fibres, two running parallel to the skin and one running first perpendicular and then parallel to the skin, representing nerve fibres connected to Merkel, Pacinian and Meissner receptors respectively. Capacitive effects, which have been found to have a minor influence on nerve activation in TENS [12], are neglected. This model is clearly an oversimplification since in practice the skin, tissue and bone have multiple compartments. However, these simplifications allow implementation of a computationally tractable model whose results are intended to approximate the real situation. Results from a model considering dermis and epidermis as separately specified layers (not presented here) were not significantly different to those obtained using the simplified single-layer skin model, as might be predicted from the work of Peters et al. [22].

**Fig 1:**
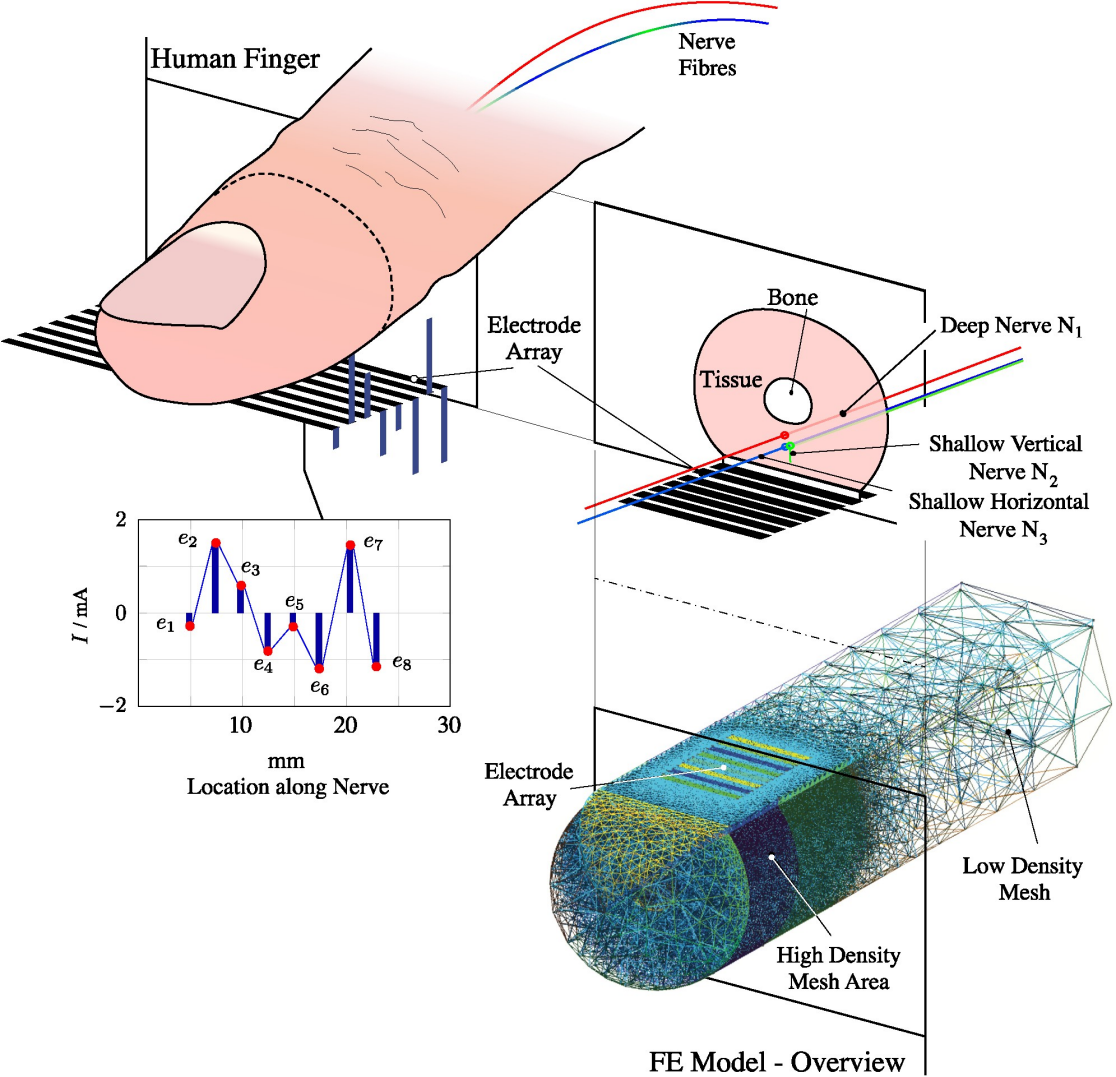
FEM developed for a human finger with an 8-electrode array located on the finger pad. A drawing of a real finger is shown at top left, with its pad on the electrode array. At top right is shown a simplified model of the finger, used to develop the FEM illustrated at bottom right (with the finger inverted to show the electrode array on the finger pad). One example of the currents flowing from array to finger is indicated in the top left image and also shown in the graph at bottom left, in which the horizontal axis represents distance along the nerve fibre, running from the fingertip towards the CNS.

The calculation of the electric potential in the FEM was achieved through the static current conduction solver with the biconjugate gradient stabilised method (BiCGStabl) and a convergence tolerance of 10^−12^. This process had to be executed once for each modelled nerve fibre. *N*_1_ was located at 1.5 mm depth and *N*_3_ at 2 mm from the skin surface (top right panel in Fig 1), both running parallel to the skin. *N*_2_ had a first portion running perpendicular to the skin from 1 to 1.5 mm depth, and then a second portion at 1.5 mm depth running parallel to the skin. The values for the finger dimensions and conductivity are listed in Tables 1 and 2. The fingernail area was connected to ground. The array of eight electrodes is modelled as a plane surface making direct contact with the finger (Fig 1). The electrode spacing is 1 mm and each electrode has dimensions 1 mm × 8.5 mm. The area covered by the current electrode design targets the majority of the receptive fields of mechanoreceptors in the human fingertip (last two thirds of the distal segment) [23]. Its dimensions are based on anthropometric data for the human index finger, which suggests that the average index fingertip measures around 16 to 22 mm in length [24, 25]. A linear array was chosen because it allows the activation of some or all of the electrodes to study the effects on fibre activation from complex stimulation patterns.

**Table 1:** Dimensions used in the finger FEM [26].

Parameter	Value / m
Finger diameter	0.02
Bone diameter	0.005
Skin thickness	0.0009
Finger length	0.084
Electrode size	0.001×0.0085

**Table 2:** Conductivity values of the human finger [27, 28].

Material	Conductivity / S/m
*σ*_*skin*_	0.0552
*σ*_*fat*_	0.0417
*σ*_*bone*_	0.0202

### 2 Nerve response model

**Fig 2:**
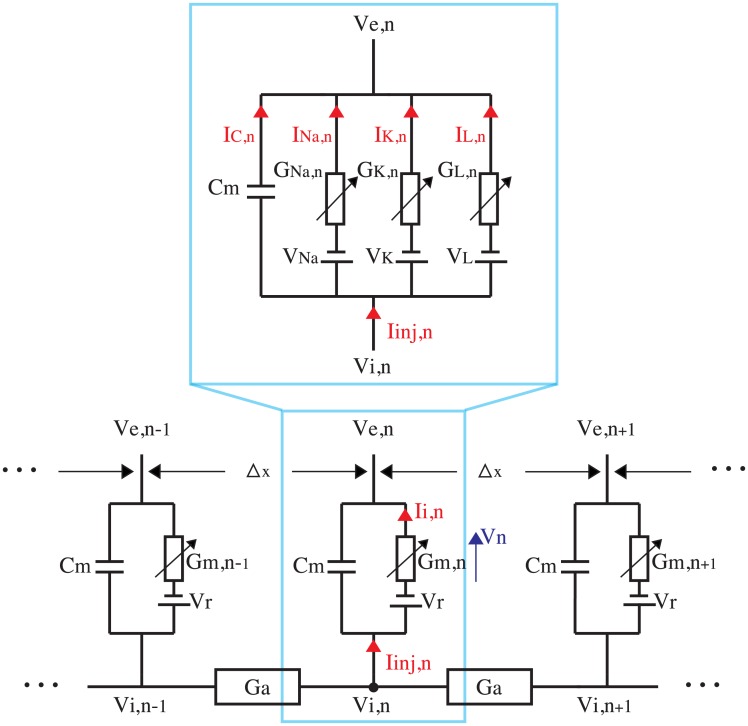
Electrical network representation of a myelinated fibre (modified from [32]).

Each of the myelinated nerve fibres is represented by an electrical cable model in which the nerve membrane is described as an electrical circuit. The nerve fibre is considered as a cylinder divided into nodes of Ranvier separated by distance Δ*x*, as shown in Fig 2, where three nodes are represented. The corresponding parameters for the nerve modelling are listed in Table 3.

**Table 3:** Variables for the electrical network representation of a myelinated fibre.

Variables	Parameter represented
*V*_*e*,*n*_	Extracellular potential at node *n*
*C*_*m*_	Membrane capacity
*G*_*m*,*n*_	Nodal membrane conductance
*G*_*a*_	Axial internodal (axoplasm) conductance
*V*_*i*,*n*_	Intracellular potential at node of Ranvier *n*
*I*_*i*,*n*_	Total ionic current
*L*	Active length of the membrane
Δx	Segment length of the fibre
*d*	Fibre diameter

Each modelled node on the nerve fibre corresponded to a group of four nodes of Ranvier, in order to reduce computational cost and simplify the analysis of the nerve response. The dynamic behaviour of each nerve node was described by the HH model using documented parameters (Tables 4 and 5, with *T* = 20°*C*) from human nerve fibres for the constants in (5) to (7). The solution of the nerve response model equations was found using MATLAB (https://uk.mathworks.com/), with two main blocks representing each nerve fibre. One block corresponds to a compartment model based on the cable model, solving (17) and (1), using the ionic current obtained through the second block, which implements the HH model, solving (14) to calculate (18). Due to the high computational cost and the stiffness of the system, the ode23s solver was selected with variable step. The excitation signal was a monophasic square pulse presented after 0.01 s with 0.00045 s width, taking into account that axon chronaxies for small myelinated fibres are generally in the range 0.0002 to 0.0007 s [29]. All the amplitudes for the electrodes were chosen within a range of −5 to 5 mA, since it is known that larger currents would result in pain and discomfort for the user, possibly leading to injuries [30, 31].

**Table 4:** HH model parameters for human nerve fibres [33–37].

Parameter	Value	*Q*_10_	*T*_0_/°*C*
Membrane resting potential *V*_*r*_	-79.4 mV	1.035	6.3
Gas constant R	8.315 J/Kmol	-	-
Faraday Constant F	9.649×10^4^ C/mol	-	-
[*Na*^+^]_*o*_/[*Na*^+^]_*i*_	7.210	-	-
[*K*^+^]_*o*_/[*K*^+^]_*i*_	0.036	-	-
[*leakage*^+^]_*o*_/[*leakage*^+^]_*i*_	0.0367	-	-
Sodium conductance *g*_*Na*_	6400 S/m^2^	1.02	24
Potassium conductance *g*_*K*_	600 S/m^2^	1.16	20
Leakage conductance *g*_*L*_	575 S/m^2^	1.418	24
Axoplasmic intracellular resistivity *ρ*_*i*_	0.25 *Ω*m	1.35^−1^	37
Membrane capacitance *C*_*m*_	0.028 F/m^2^	-	-
Fibre diameter *d*	4 *μ*m	-	-
Distance between nodes of Ranvier Δ*x*	78.461 *μ*m	-	-
Nodal length *L*	1.061 *μ*m	-	-

**Table 5:** HH model parameters for human nerve fibres [33].

Parameter	*Q*_10_	*T*_0_/°*C*	A	B	C	D
*α*_*m*_	2.23	6.3	4.42	2.5	0.1	1
*β*_*m*_	2.23	6.3	4.42	4.0	18	-
*α*_*n*_	1.5	6.3	1.47	0.07	20	-
*β*_*n*_	1.5	6.3	1.47	3.0	0.1	-
*α*_*h*_	1.5	6.3	0.2	1.0	0.1	10
*β*_*h*_	1.5	6.3	0.2	0.125	80	-

The effect of each electrode current regarding a possible cathodic block of the nerve fibre is of particular interest here. The Frankenhaeuser-Huxley (FH) equations, generally used to describe myelinated fibres, do not allow simulation of such a block [38]. Hence, the Hodgkin and Huxley (HH) equations, which can simulate a cathodic block, were chosen for use in the model (they are normally used to represent unmyelinated fibres, but are here implemented with the corresponding modifications to describe a myelinated fibre [33]). A single node can be locally represented by the HH equations [8]. The expanded circuit at the top of Fig 2 represents one node. It shows how the membrane conductance *G*_*m*,*n*_ of a node derives from the leakage conductance *G*_*L*,*n*_ representing ion diffusion through the membrane, and from the sodium and potassium conductances, *G*_*Na*,*n*_ and *G*_*K*,*n*_, dependent on the particularities of each channel and on the probability of it being open. The injected membrane current at the *n*th node *I*_*inj*,*n*_ is the sum of the currents flowing through the capacitor *C*_*m*_ and the membrane conductance *G*_*m*,*n*_ (for all the equations used to develop this section, see Appendix).

## Results

In the following subsections we present simulation results using the aforementioned model for an overall validation and the study of two cases of interest with respect to selective nerve stimulation. The first one corresponds to an experimentally documented case by Yem and Kajimoto [39]; their results suggest that cathodic stimulation excites fibres from both Merkel cells (running parallel to the skin) and Meissner corpuscles (running first perpendicular to the skin and then parallel to the skin), whereas anodic stimulation excites fibres from Meissner corpuscles only. The second scenario consists of two nerve fibres running parallel to the skin at different depths, simulating fibres connected to Merkel and Pacinian receptors, that are selectively stimulated through a specific pattern of injected currents. Both scenarios are used as examples for demonstrating the usage of the simulation environment in the context of two important steps of studying tactile perception: the mathematical modelling of observed experimental findings (case 1) and the formulation of new hypotheses (case 2), which form the basis for new experimental studies.

### 1 Overall validation of simulation environment

To illustrate the performance of the simulation model we provide simulation results of a two and eight active electrode setup using one nerve fibre running parallel to the skin at constant depth. For both setups we show the effects evaluated at two stages, as proposed by McNeal [7]:

1. The mapping of the currents *I*_*el*_ applied through the electrode array to the extracellular voltage *V*_*e*,*n*_, evaluating the FEM.
2. The mapping of the extracellular voltage *V*_*e*,*n*_ to the membrane potential of a specific nerve fibre *V*_*n*_, involving the link between the FEM and the nerve fibre model.

#### 1.1 Two-electrode setup

**Fig 3:**
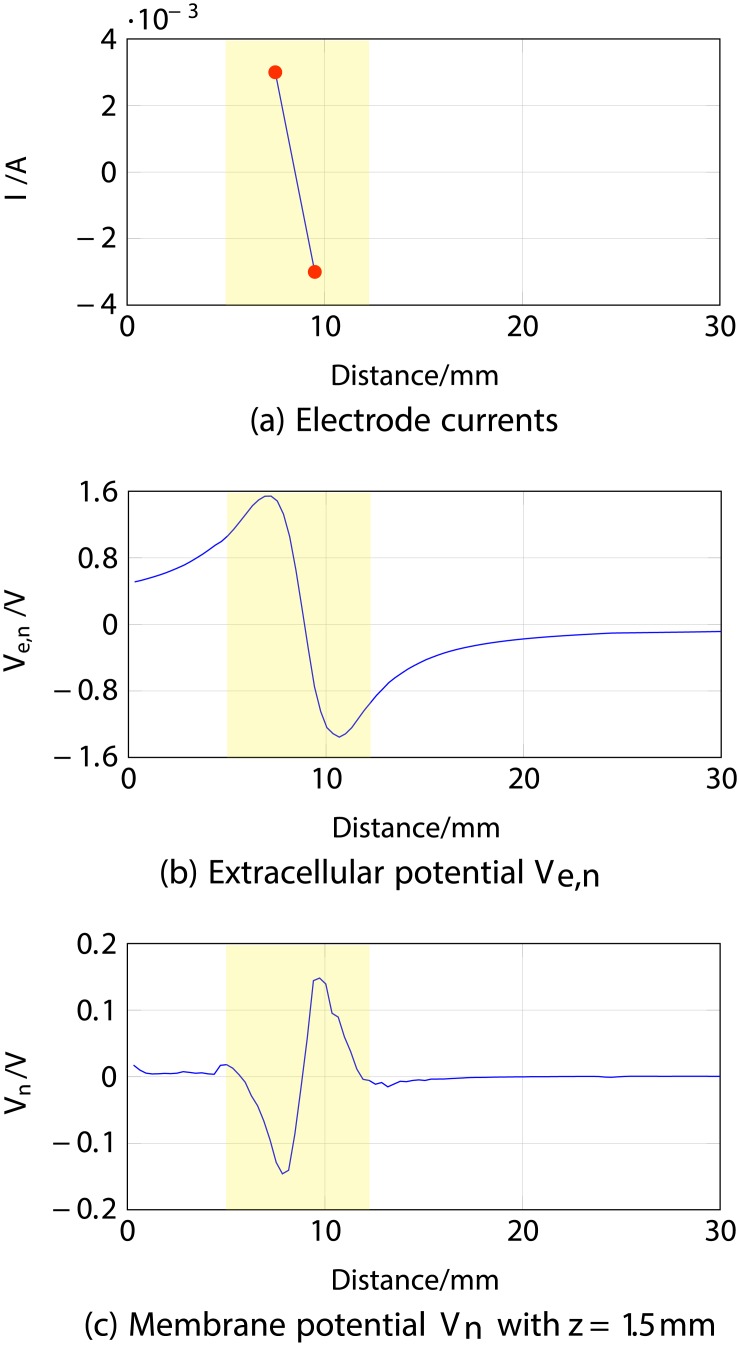
ES simulation of a nerve fibre located at 1.5 mm depth using two electrodes, 1 ms after stimulus onset. *a*) corresponds to the electrode currents, *b*) to the extracellular potential *V*_*e*,*n*_ and *c*) to the membrane potential *V*_*n*_ with depth *z* = 1.5 mm.

The nerve fibre was modelled at depth *z* = 1.5 mm with two transcutaneous electrodes located at 7 and 9 mm from the fingertip (panel *a*) in Fig 3), with currents *I*_*el*,1_ = 3 mA and *I*_*el*,2_ = −3 mA. Panel *b*) shows the results of stage one in form of the extracellular potential *V*_*e*,*n*_ as a function of distance (millimetres) along the nerve fibre.

Since the exact position of the electrodes with respect to the nerves is known, the tracing of the change in voltage resulting from the electrode currents is straightforward. The extracellular potential *V*_*e*,*n*_ plot shows the expected result (proximity to the anodic stimulation, i.e. positive currents, increasing the membrane potential *V*_*e*,*n*_ and proximity to cathodic stimulation decreasing *V*_*e*,*n*_) in the area of interest (marked in yellow in panels *a*), *b*) and *c*) in Fig 3).

The membrane potential is shown as a reduced voltage *V*_*n*_ (i.e., the static offset is subtracted); thus, positive values of the potential represent the fibre’s depolarisation, and negative hyperpolarisation. It can be seen from Fig 3 that there is a correspondence between the curves for *V*_*n*_ and the *V*_*e*,*n*_, where negative currents produce the excitation of the fibre and positive currents an inhibition.

#### 1.2 Eight-electrode setup

This simulation involved the investigation of the behaviour of the same myelinated nerve fibre (depth *z* = 1.5 mm) using all electrodes in the eight-electrode array. The electrode currents *I*_*el*,1_ to *I*_*el*,8_ were -0.43, -0.453, 0.36, 0.23, -0.024, 0.36, -0.047 and -0.004 mA. These values were randomly created using a uniform distribution within the interval (−5,5) mA rejecting patterns whose sum was not approximately zero (taking into consideration the safety constraint), thus using more likely low currents than high currents. This ensures that no currents will flow deeply in the human body, which can cause tissue damage [40]. In the FEM, the overall effect of the eight-electrode array is determined using superposition.

**Fig 4:**
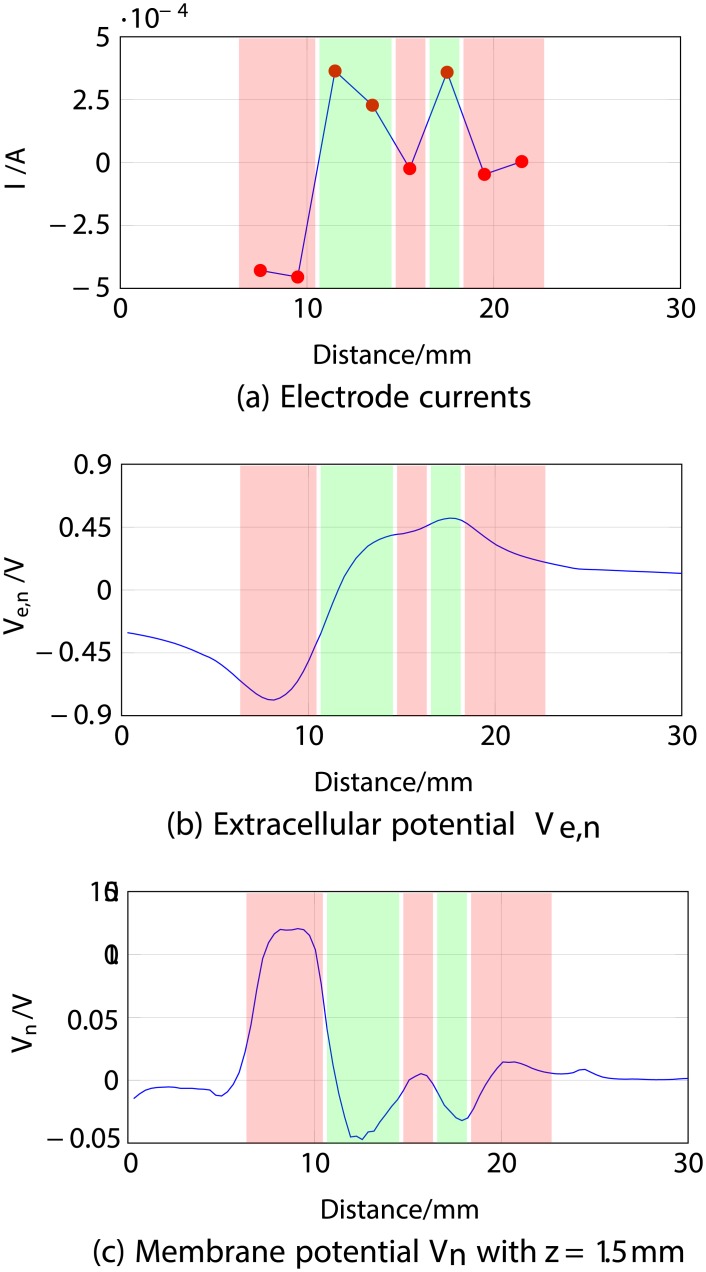
ES simulation of a nerve fibre located at 1.5 mm depth using eight electrodes, 1 ms after stimulus onset. *a*) corresponds to the electrode currents, *b*) to the extracellular potential *V*_*e*,*n*_ and *c*) to the membrane potential *V*_*n*_ with depth *z* = 1.5 mm.

The stated stimulus and the generated response are shown in Fig 4, from which it may again be observed that proximity to an anodic stimulation (from the third, fourth and sixth electrodes) is associated with an increase in the extracellular potential *V*_*e*,*n*_ and a hyperpolarisation of the nerve fibre (green areas from 11 to 14 mm and 16 to 18 mm). Equivalently, proximity to a cathodic stimulation (from the other five electrodes) decreases *V*_*e*,*n*_, depolarising the fibre (red areas covering the first, second, fifth, seventh and eighth electrode). The modelled membrane potential again matches *V*_*e*,*n*_.

### 2 Selective stimulation

Our first analysed case is based on the aforementioned experimental findings by Yem and Kajimoto [39], and aims at demonstrating that the present simulation environment has a sufficient level of detail to replicate their experimental results. Yem and Kajimoto showed in their experiments that an anodic stimulation mainly produced a vibration sensation, and that a cathodic stimulation provided both vibration and pressure sensations [39]. Physiological findings indicate that Merkel cells respond to vibration and Meissner corpuscles to pressure, and that the nerves connected to Merkel cells run parallel to the skin and nerves connected to Meissner corpuscles run firstly perpendicular to the skin before changing to a parallel orientation [41, 42]. Following these assumptions, one nerve fibre (*N*_1_) is simulated to run parallel to the skin at 1.5 mm depth (representing a fibre from a Merkel cell) and a second nerve fibre (*N*_2_) is simulated to run perpendicular to the skin from 1 to 1.5 mm depth and then parallel to the skin at 1.5 mm depth (representing a fibre from a Meissner corpuscle) using eight active electrodes. *N*_2_ was located directly under the fourth electrode.

Further, and motivated by experiments performed by Kajimoto et al. that showed selective stimulation of three different types of mechanoreceptors [17], we also simulate a second case consisting of two nerve fibres running parallel to the skin located at 1.5 mm depth (*N*_1_) and 2 mm depth (*N*_3_), representing nerve fibres from a Merkel and a Pacinian receptor (assuming that fibres from Pacinian receptors run parallel to the skin, deeper than those from Merkel and Meissner receptors [42]). The main objective of this scenario (using eight active electrodes) was to check if the present simulation environment provides similar results when compared to the experimental findings by Kajimoto et al. [17]. The response of all fibres was traced in distance and time.

#### 2.1 Selective stimulation with a parallel and a perpendicular nerve fibre

Anodic or cathodic stimulation requires a small electrode to deliver the stimulation current and a large electrode to provide the return current path [39]. Thus, using the 8-electrode system outlined above (Fig 1), the fourth electrode was set as the main stimulation point and the other seven electrodes provided the return path, effectively acting as a larger return electrode.

**Fig 5:**
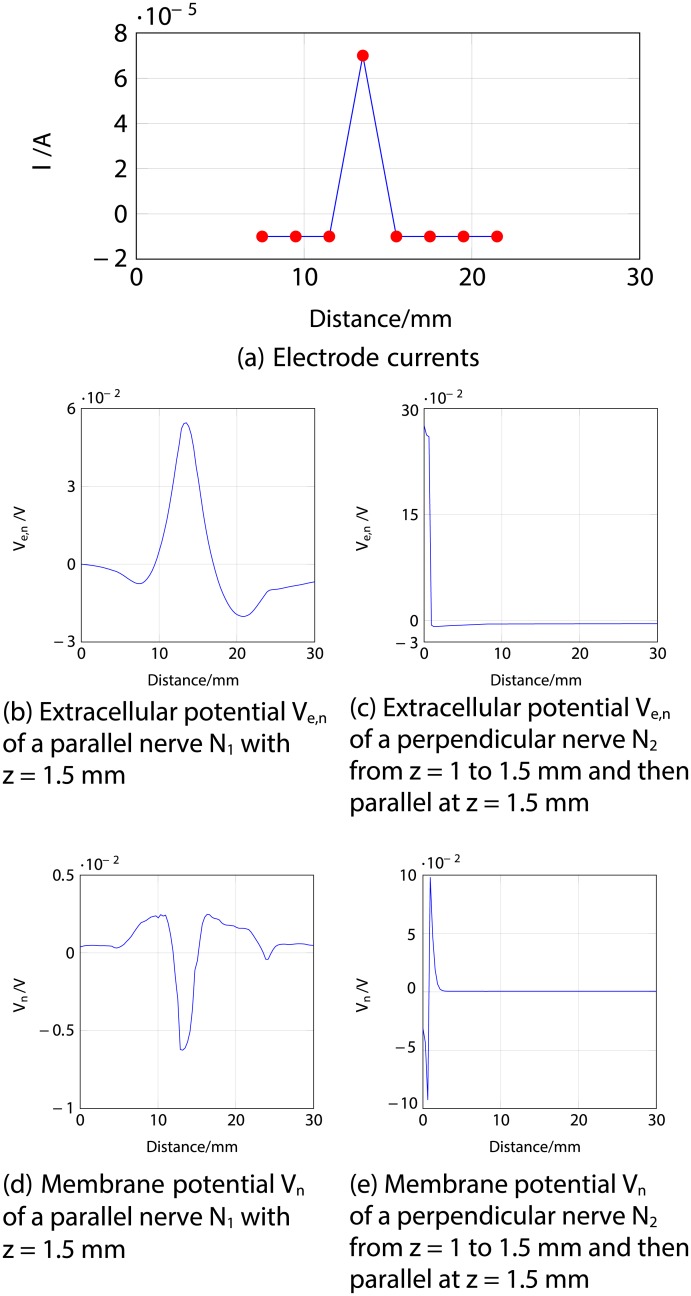
Anodic ES simulation of a nerve fibre running parallel to the skin at 1.5 mm depth (*N*_1_) and a nerve fibre running perpendicular from 1 to 1.5 mm depth and then parallel to the skin at 1.5 mm depth (*N*_2_) at 1 ms after stimulus onset. *a*) corresponds to the electrode currents, *b*) and *c*) to the extracellular potentials *V*_*e*,*n*_ and *d*) and *e*) to the membrane potentials *V*_*n*_ as functions of distance (millimetres). Figure shows the efficient depolarisation of *N*_2_ and the inhibition of *N*_1_. In panels *b*), *c*), *d*) and *e*), the horizontal axis represents distance along the nerve, for the parallel nerve shown in *b*) and *d*), distance along the nerve corresponds to distance along the skin, as in panel *a*); but for the perpendicular nerve in panels *c*) and *e*), which originates under the 4th electrode and runs first perpendicular to and then parallel to the skin, distance along the nerve is offset with respect to distance along the skin in *a*).

**Fig 6:**
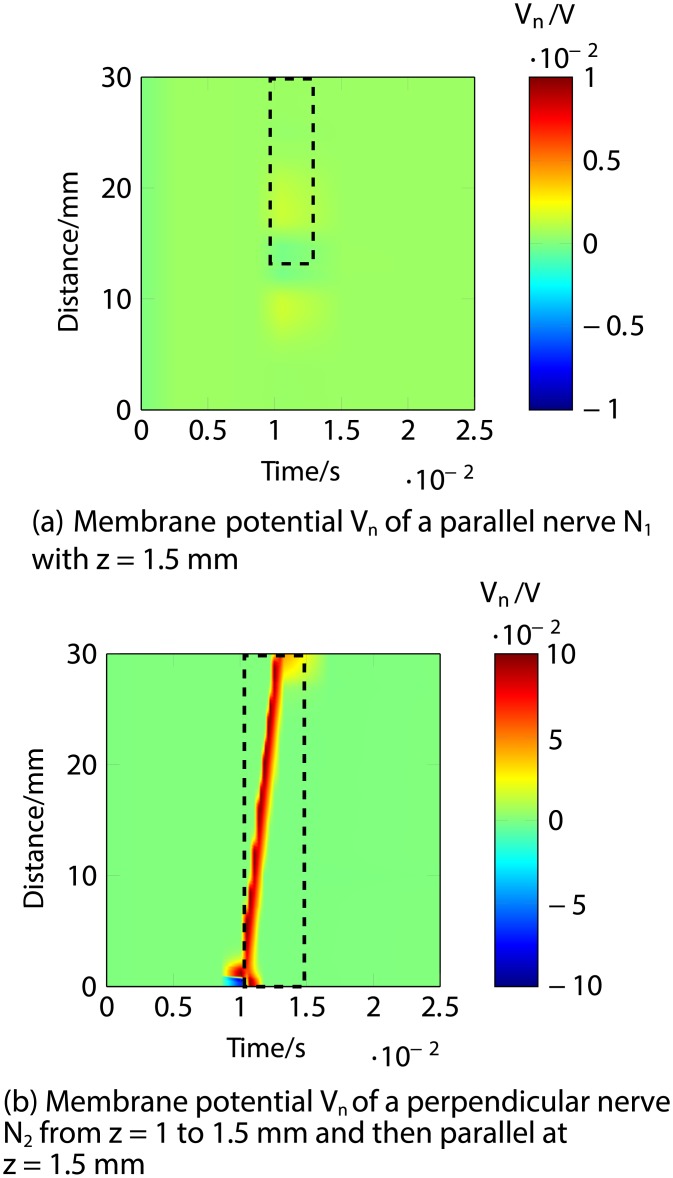
Anodic ES simulation of a nerve fibre running parallel to the skin at 1.5 mm depth (*N*_1_) and a nerve fibre running perpendicular from 1 to 1.5 mm depth and then parallel to the skin at 1.5 mm depth (*N*_2_) showing the responses through time. *a*) shows the lack of an action potential in the membrane potential *V*_*n*_ of *N*_1_ (hence considered inhibited). *b*) corresponds to the membrane potential *V*_*n*_ of *N*_2_, highlighting the excitation and travelling of the spike towards the end of the modelled nerve fibre (propagating towards the CNS, thus considered activated).

**Fig 7:**
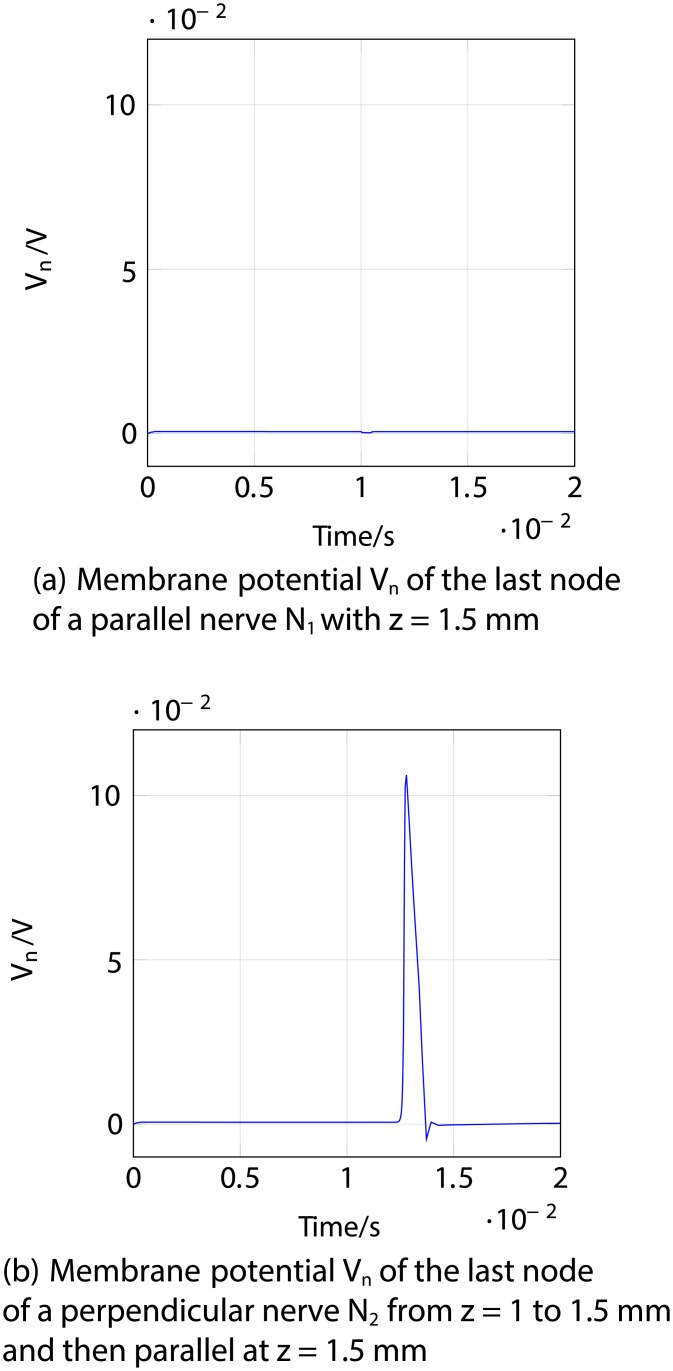
Anodic ES simulation of a nerve fibre running parallel to the skin at 1.5 mm depth (*N*_1_) and a nerve fibre running perpendicular from 1 to 1.5 mm depth and then parallel to the skin at 1.5 mm depth (*N*_2_) showing the response of the fibres in the last node (end towards the CNS) through time. *a*) shows the lack of an action potential in the last node of the membrane potential *V*_*n*_ of *N*_1_ (hence considered inhibited). *b*) corresponds to the membrane potential *V*_*n*_ of the last node of *N*_2_, where the shape of the action potential is shown, thus indicating the activation of the fibre due to the propagation of the spike towards the CNS.

**Fig 8:**
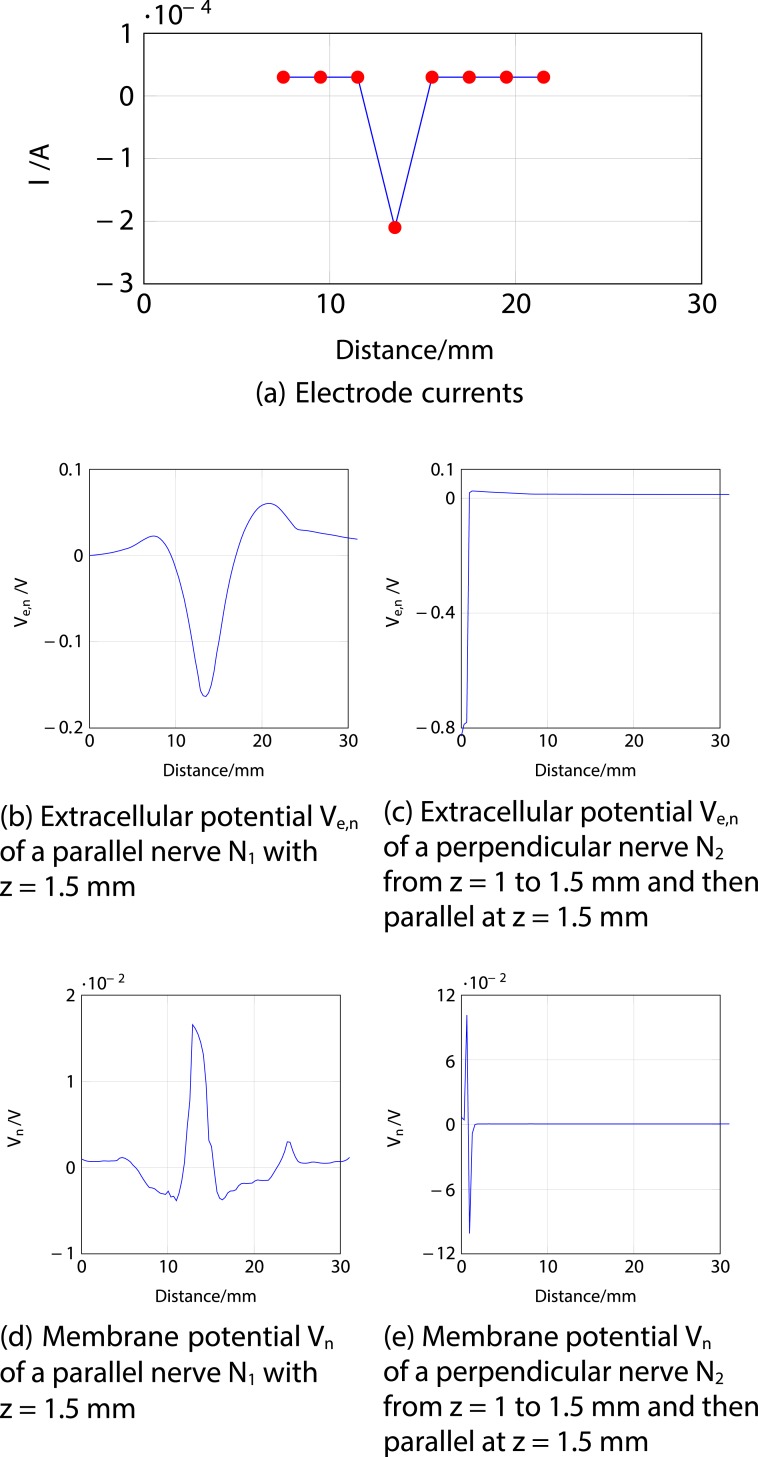
Cathodic ES simulation of a nerve fibre running parallel to the skin at 1.5 mm depth (*N*_1_) and a nerve fibre running perpendicular from 1 to 1.5 mm depth and then parallel to the skin at 1.5 mm depth (*N*_2_) at 1 ms after stimulus onset. *a*) corresponds to the electrode currents, *b*) and *c*) to the extracellular potentials *V*_*e*,*n*_ and *d*) and *e*) to the membrane potentials *V*_*n*_ as functions of distance (millimetres). Figure shows the depolarisation of *N*_1_ and *N*_2_. In panels *b*), *c*), *d*) and *e*), the horizontal axis represents distance along the nerve, for the parallel nerve shown in *b*) and *d*), distance along the nerve corresponds to distance along the skin, as in panel *a*); but for the perpendicular nerve in panels *c*) and *e*), which originates under the 4th electrode and runs first perpendicular to and then parallel to the skin, distance along the nerve is offset with respect to distance along the skin in *a*).

**Fig 9:**
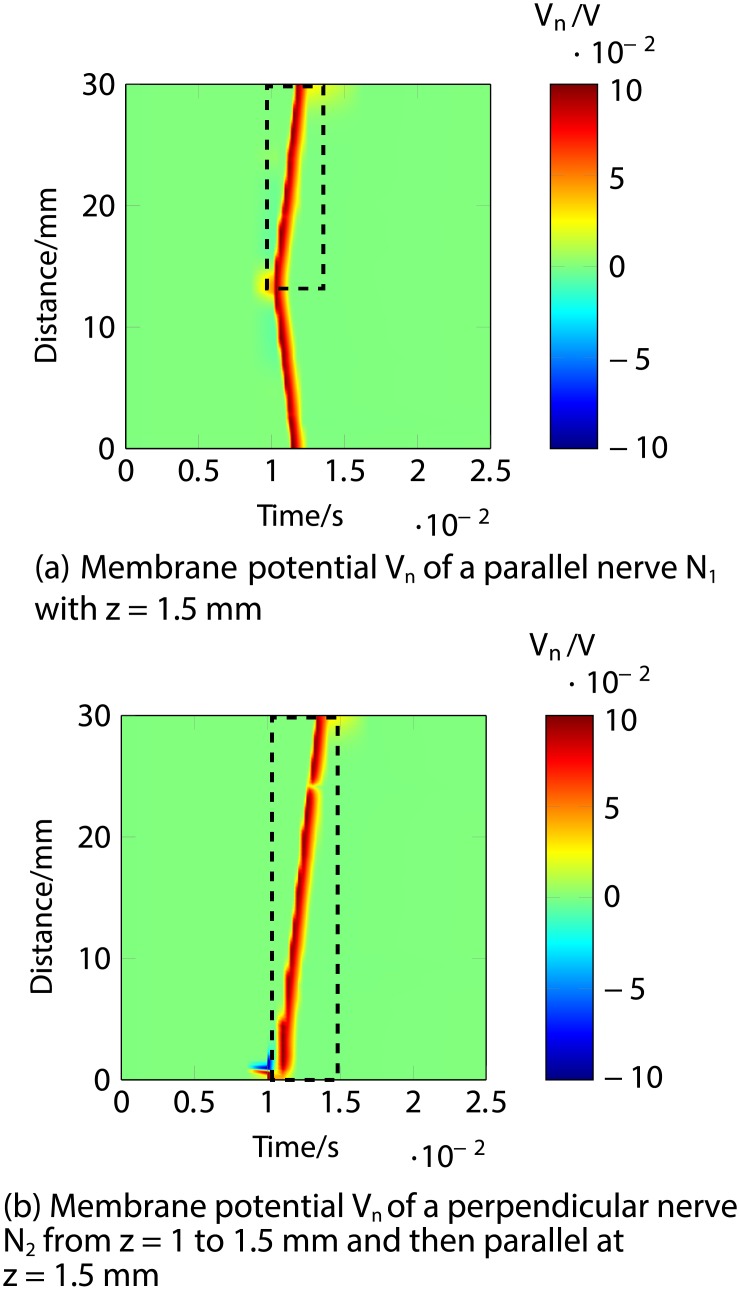
Cathodic ES simulation of a nerve fibre running parallel to the skin at 1.5 mm depth (*N*_1_) and a nerve fibre running perpendicular from 1 to 1.5 mm depth and then parallel to the skin at 1.5 mm depth (*N*_2_) showing the responses through time. *a*) corresponds to the membrane potential *V*_*n*_ of *N*_1_ and *b*) to the membrane potential *V*_*n*_ of *N*_2_. The excitations and the traveling of the spikes towards the end of both nerve fibres (towards the CNS, hence considered activated) are highlighted.

**Fig 10:**
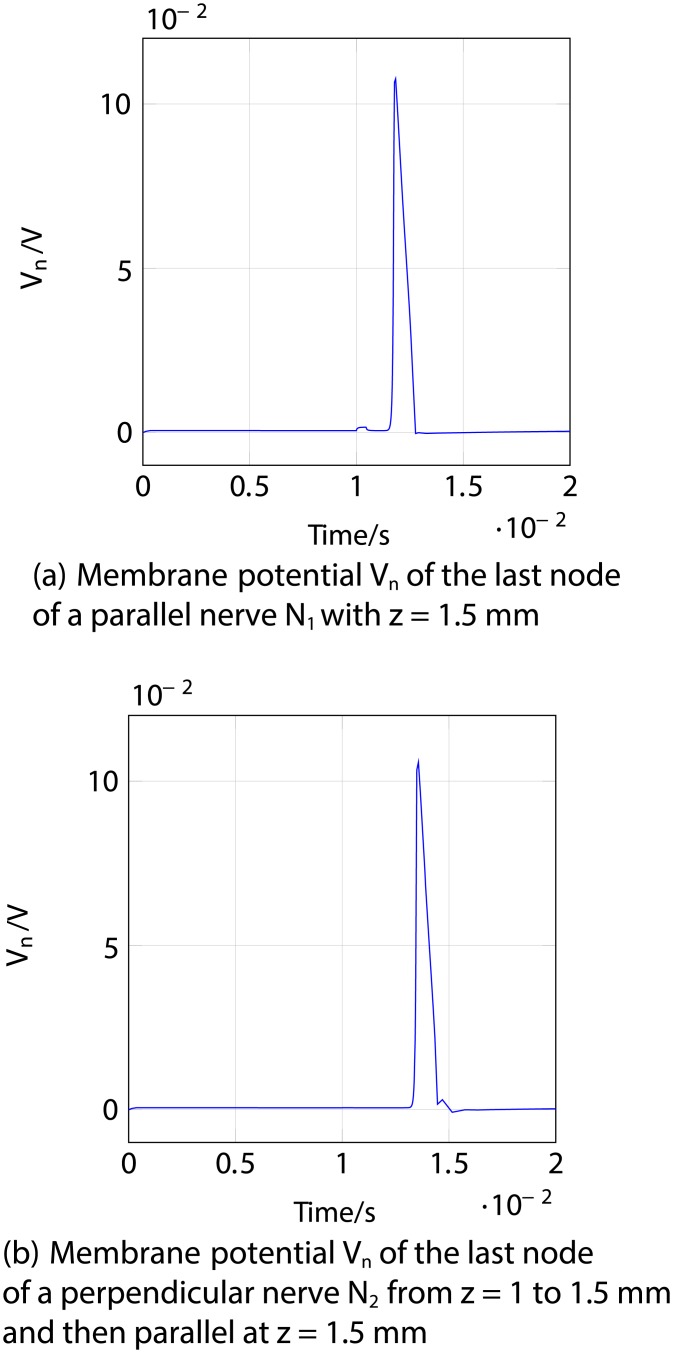
Cathodic ES simulation of a nerve fibre running parallel to the skin at 1.5 mm depth (*N*_1_) and a nerve fibre running perpendicular from 1 to 1.5 mm depth and then parallel to the skin at 1.5 mm depth (*N*_2_) showing the responses of the fibres in the last node (end towards the CNS) through time. *a*) corresponds to the membrane potential *V*_*n*_ of the last node of *N*_1_ and *b*) to the membrane potential *V*_*n*_ of the last node of *N*_2_. In both panels it can be seen an action potential, which denotes the activation of the fibres.

Firstly, an anodic stimulation was simulated by setting the electrode currents *I*_*el*,1_ to *I*_*el*,8_ to -0.01, -0.01, -0.01, 0.07, -0.01, -0.01, -0.01 and −0.01 mA (panel *a*) in Fig 5). The extracellular potential *V*_*e*,*n*_ is illustrated in *b*) and *c*) as a function of distance (millimetres) along the parallel nerve fibre (panel *b*) in Fig 5) and perpendicular nerve fibre (panel *c*) in Fig 5). The membrane potential *V*_*n*_ is shown in *d*) and *e*) as a function of distance along *N*_1_ (panel *d*) in Fig 5) and *N*_2_ (panel *e*) in Fig 5). The results fit to the experimental findings of Yem and Kajimoto [39], showing the anodic stimulation to activate the perpendicular fibre *N*_2_ and to inhibit the parallel fibre *N*_1_, as depicted in *d*) and *e*) in Fig 5. *N*_2_ was considered activated due to the propagation of the excitation along the perpendicular and parallel portions of the nerve fibre towards the CNS, as shown in *b*) in Figs 6 and 7. Similarly, *a*) in Figs 6 and 7 show that there is no spike propagation along the parallel fibre *N*_1_ (thus, it is considered inhibited). Fig 7 illustrates the shape of the action potential generated in *N*_2_ in the last node, denoting its propagation towards the CNS (categorising the fibre as activated). The second setup used 0.03, 0.03, 0.03, -0.21, 0.03, 0.03, 0.03 and 0.03 mA for the electrode currents *I*_*el*,1_ to *I*_*el*,8_, presenting a cathodic stimulation around the fourth electrode (panel *a*) in Fig 8). The extracellular potential *V*_*e*,*n*_ is displayed in *b*) and *c*) as a function of distance (millimetres) along the parallel fibre *N*_1_ (panel *b*) in Fig 8) and the perpendicular fibre *N*_2_ (panel *c*) in Fig 8), and the membrane potential *V*_*n*_ is shown in *d*) and *e*) as a function of distance along *N*_1_ (panel *d*) in Fig 8) and *N*_2_ (panel *e*) in Fig 8). The responses are also consistent with Yem and Kajimoto’s experimental results [39], showing the activation of both fibres (Figs 9 and 10). Fig 10 illustrates the shape of the action potentials generated in *N*_1_ and *N*_2_ in the last node, denoting their propagation towards the CNS (categorising the fibres as activated).

#### 2.2 Selective stimulation with two parallel nerve fibres

The effect of different excitation patterns was determined by testing 1000 randomised patterns for the injected currents. The patterns were generated using a uniform distribution within the interval (−5,5) mA, rejecting patterns whose sum was not approximately zero (taking into consideration the safety constraint), thus using more likely low currents than high currents. A nerve activation was considered to be valid if the excitation propagated to that end of the fibre (at the location labelled 30 mm) which represents a connection to the CNS. Likewise, a nerve was considered inhibited when no action potential was found in the last node (end towards the CNS).

The selective stimulation of the shallower nerve *N*_1_ was achieved in three main scenarios: from the 1000 patterns, five tests showed that the stimulus was not sufficient to produce a significant excitation in *N*_3_ (the fibre showed minimal change in its membrane potential), but *N*_1_ was activated; 20 tests with the last electrode injecting a positive current produced an action potential in *N*_1_, but resulted in a negative membrane potential in *N*_3_ which inhibited excitation of the fibre; and five tests with the last electrode injecting a negative current showed a cathodic block in *N*_3_ and an action potential in *N*_1_ as an “overshoot” [38] of the cathodic stimulation.

**Fig 11:**
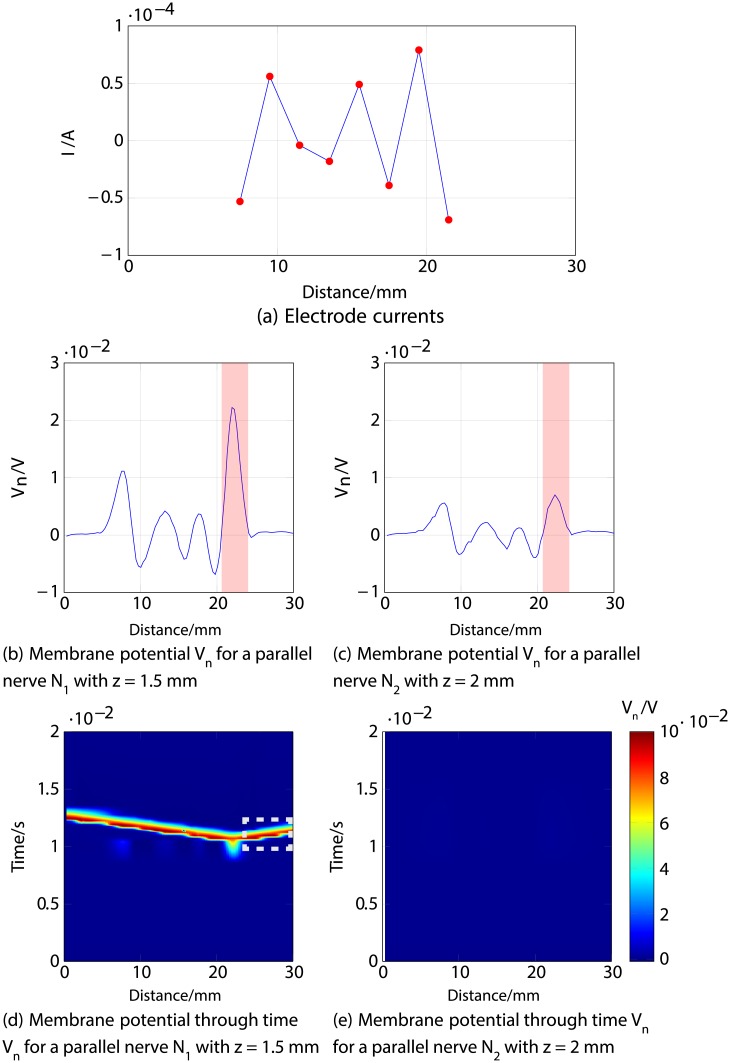
First example of selective stimulation of the shallower nerve fibre *N*_1_. *a*) shows the electrode currents. *b*) and *c*) illustrate the membrane potential *V*_*n*_ of *N*_1_ and *N*_3_, respectively, 1 ms after stimulus onset. *d*) and *e*) correspond to the time courses of the excitations; it can be seen (region indicated by dotted lines) that an excitation (shown in red) propagates towards the nerve ending (CNS) in *N*_1_ (thus considered activated), as shown in *d*), but not in *N*_3_ illustrated in *e*) (thus considered inhibited).

**Fig 12:**
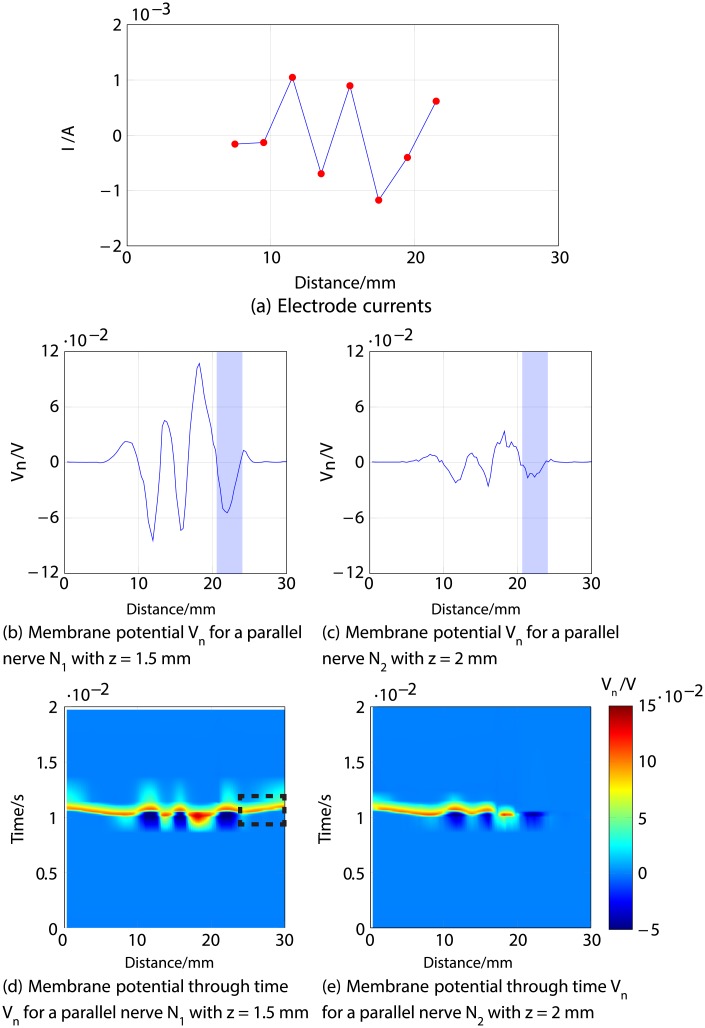
Second example of selective stimulation of the shallower nerve fibre *N*_1_. *a*) shows the electrode currents. *b*) and *c*) illustrate the membrane potential *V*_*n*_ of *N*_1_ and *N*_3_, respectively, 1 ms after stimulus onset. *d*) and *e*) correspond to the time courses of the excitations; it is shown in the region indicated by dotted lines in *d*), that an excitation (shown in red) propagates towards the nerve ending (CNS) in *N*_1_ (thus considered activated), but not in *N*_3_, illustrated in *e*) (thus considered inhibited).

**Fig 13:**
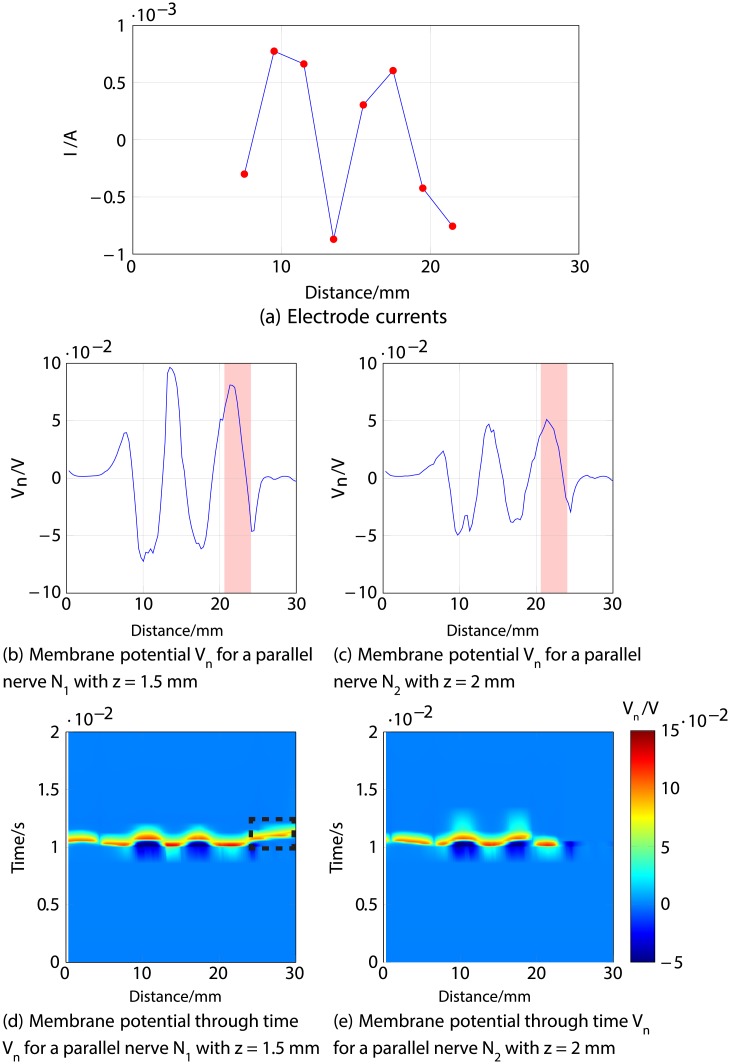
Third example of selective stimulation of the shallower nerve fibre *N*_1_. *a*) shows the electrode currents. *b*) and *c*) illustrate the membrane potential *V*_*n*_ of *N*_1_ and *N*_3_, respectively, 1 ms after stimulus onset. *d*) and *e*) correspond to the time courses of the excitations; the region indicated by dotted lines in *d*) demonstrates that an excitation (shown in red) propagates towards the nerve ending (CNS) in *N*_1_ (thus considered activated), but not in *N*_3_, illustrated in *e*) (thus considered inhibited).

Figs 11, 12 and 13 describe an example for each scenario. For all three cases, Figs 11, 12 and 13 show the response of both nerve fibres, illustrating the applied currents the modelled membrane potential in the nodes, showing an action potential propagating towards the end of the fibre (around 30 mm) in all cases for *N*_1_ (thus considered activated), and no excitation in *N*_3_ (considered inhibited).

In Fig 11 a positive membrane potential is observed in both fibres *N*_1_ and *N*_3_ around 21 to 23 mm, highlighted in red in panels *b*) and *c*), deriving from the negative current at the last electrode. However, this excitation results in an action potential only in *N*_1_, which is shown in panel *d*) in Fig 11, propagating towards the end of the nerve (CNS). *N*_3_ is classified as inhibited as a result of the lack of action potential travelling to the CNS, as observed in panel *e*).

The second example for selective stimulation of *N*_1_ is described in Fig 12, which shows that the positive current at the last electrode produces a negative membrane potential in both fibres around 21 to 23 mm, highlighted in blue in panels *b*) and *c*), together with an adjacent positive “overshoot” [38] in the shallower nerve *N*_1_ around 24 mm. This results in an inhibition (no action potential) of *N*_3_ (depicted in panel *e*) in Fig 12), but the positive membrane potential in *N*_1_ originates an action potential that travels towards the CNS, detailed in panel *d*).

Fig 13 corresponds to the last case of selective stimulation of *N*_1_, where a positive membrane potential is observed in both fibres *N*_1_ and *N*_3_ around 18 to 22 mm, highlighted in red in panels *b*) and *c*), together with an adjacent negative “overshoot” [38] at around 24 mm. These features derive from the negative current at the last two electrodes. As a result, *N*_3_ shows a cathodic block (no action potential is propagating towards the CNS, as depicted in panel *d*)), but a positive membrane potential is generated in *N*_1_, producing an action potential propagating towards the end of the nerve (CNS), shown in panel *e*).

**Fig 14:**
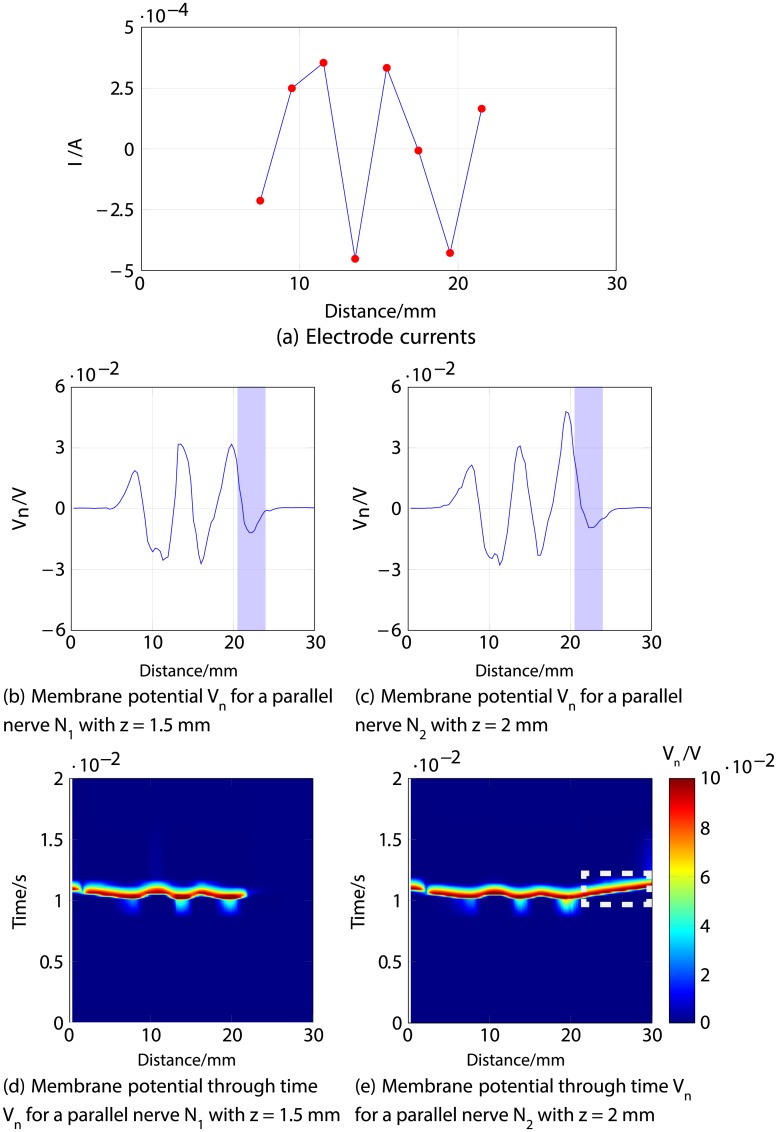
First example of selective stimulation of the deeper nerve fibre *N*_3_. *a*) shows the electrode currents. *b*) and *c*) illustrate the membrane potential *V*_*n*_ of *N*_1_ and *N*_3_, respectively, 1 ms after stimulus onset. *d*) and *e*) correspond to the time courses of the excitations; it can be seen (region indicated by dotted lines) that an excitation (shown in red) propagates towards the nerve ending (CNS) in *N*_3_ as shown in *e*) (thus considered activated), but not in *N*_1_ illustrated in *d*) (thus considered inhibited).

**Fig 15:**
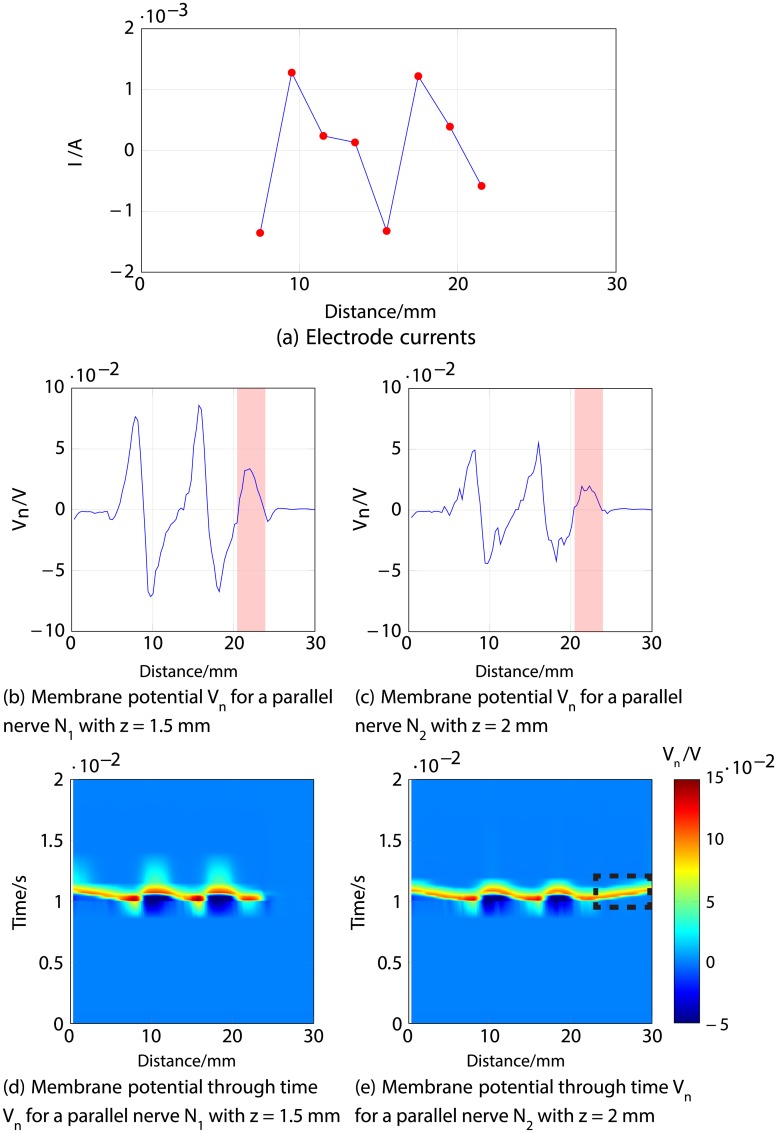
Second example of selective stimulation of the deeper nerve fibre *N*_3_. *a*) shows the electrode currents. *b*) and *c*) illustrate the membrane potential *V*_*n*_ of *N*_1_ and *N*_3_, respectively, 1 ms after stimulus onset. *d*) and *e*) correspond to the time courses of the excitations; the region indicated by dotted lines in *d*) shows that an excitation (in red) propagates towards the nerve ending (CNS) in *N*_1_ (thus considered activated), but not in *N*_3_, depicted in *e*) (thus considered inhibited).

Regarding the selective stimulation of *N*_3_, two scenarios were observed, both involving inhibition of all excitations in *N*_1_, but not in *N*_3_: 23 cases out of the 1000 tests were found with the last electrode injecting a positive current (producing a negative potential in both fibres that stopped any excitation generated in *N*_1_, but was not sufficient to stop the travelling of the action potential generated in *N*_3_ from the previous electrodes); and two cases with the last electrode injecting negative current (inducing a cathodic block in *N*_1_, stopping the excitation of the fibre, but the cathodic stimulation was not sufficient to stop the action potential generated in *N*_3_). Examples of the two scenarios are illustrated in Figs 14 and 15, clearly showing an action potential in all cases for the last node in *N*_3_ (thus considered activated), and no activation in *N*_1_ (thus considered inhibited).

In Fig 14 a negative membrane potential is observed in both fibres *N*_1_ and *N*_3_ around 21 to 23 mm, highlighted in blue in panels *b*) and *c*), deriving from the positive current at the last electrode. This results in an inhibition (the action potential generated from the previous electrode is stopped) only in *N*_1_, shown in panel *d*); while the action potential in *N*_3_ continues to propagate towards the end of the nerve, as depicted in panel *e*).

The second example for selective stimulation of *N*_3_ is shown in Fig 15, where a positive membrane potential is observed in both fibres *N*_1_ and *N*_3_ around 21 to 23 mm, highlighted in red in panels *b*) and *c*), together with an adjacent “overshoot” [38] at around 24 mm in *N*_1_. These features derive from the negative current at the last electrode. The hyperpolarisation in *N*_1_ stops the action potential from propagating towards the CNS, as observed in panel *d*) in Fig 15; the corresponding hyperpolarisation in *N*_3_ is minimal, and not strong enough to prevent an action potential from travelling to the end of the nerve, as shown in panel *e*).

A fibre can be activated with either a single cathodic or anodic stimulation [38], and the modelling results suggest that the selective stimulation of a specific parallel fibre is mostly dependent on the stimulation provided by the last two electrodes. This suggests that similar excitations to those described above might be produced using less than eight electrodes. To investigate this, two trials were run, modifying the currents used for the 1000 tests so that only the last three or the last two electrodes were activated; i.e., the rest of the electrodes carried no current. With three electrodes, the seventh and eighth electrodes kept their original current values, and the sixth electrode carried a current to balance these two; similarly, with two electrodes, the eighth electrode kept its original current value, and the seventh electrode carried the inverse, to balance this. Results showed that it was indeed possible to selectively stimulate either fibre using fewer active electrodes. However, the number of cases of interest (selective activation of *N*_3_) dropped as the number of electrodes was reduced. When using three active electrodes targeting the selective activation of *N*_1_, 14 cases produced no significant response in *N*_3_ (as in Fig 11), 12 cases presented an anodic stimulation (as in Fig 12) and 7 a cathodic stimulation (as in Fig 13). For stimulating *N*_1_ with two electrodes, 37 cases produced no significant response in *N*_3_, 6 cases had an anodic stimulation and 8 a cathodic stimulation. For selective activation of *N*_3_ with three electrodes, 15 cases showed an anodic stimulation (as in Fig 14) and 4 cases a cathodic stimulation (as in Fig 15). Targeting *N*_3_ with two electrodes, 5 cases had an anodic stimulation and 8 cases a cathodic stimulation.

## Discussion

The results from the present study show that by suitable choice of electrode currents a specific nerve fibre can be selectively stimulated.

Regarding the scenario with one parallel fibre *N*_1_ and one perpendicular fibre *N*_2_, simulation results were found to support experimental findings [39], indicating that the chosen level of complexity of the model is sufficient to capture such effects. For the cathodic stimulation, it was necessary to use currents three times greater than the currents for the anodic stimulation. This is due to the difference between the thresholds for the excitations (for the case of fingertip skin, sensation thresholds for anodic stimulation have been found to be lower than for cathodic stimulation [43]).

For the case of two parallel fibres, *N*_1_ and *N*_3_, it has been demonstrated that stimulation currents can be chosen to excite only one of the two fibres and inhibit the other. Such selectivity was not achieved in previous studies by Kajimoto [16, 39], where stimulation of shallower fibres was always observed when deeper fibres were targeted. In fact, such unwanted stimulation of shallower fibres was observed in more than 90% of the random stimulation patterns tested in the present study; however, selective stimulation of the deeper nerve fibre (inhibiting the shallower nerve fibre) was possible in over 2% of cases, with appropriate stimulation patterns. To provide an explanation for this, it is necessary to look for common features in the subset of the random stimulation patterns that is associated with selective stimulation.

Inspection of the modelled responses indicated that a fibre was activated by a stimulus which produced a positive membrane potential in approximately 10 consecutive nodes, or more. Similarly, a fibre was inhibited (stopping any previous action potential) by a stimulus which produced a negative membrane potential in at least 15 consecutive nodes (with either cathodic or anodic stimulation). As might be expected, the effectiveness of the stimulus was found to vary with the depth of the modelled fibres.

Since the excitation patterns which achieved selective stimulation did not at first sight hold clear commonalities, it was necessary to scrutinise the responses of the modelled fibres to look for shared characteristics. As supported by our simulations, there are different situations that produce selective excitation of the shallower nerve fibre *N*_1_: a relatively weak stimulation excites *N*_1_ but not *N*_3_ (Fig 11), or a stronger stimulation (anodic or cathodic) produces activation and inhibition in both nerve fibres, with a residual (final excitation) in *N*_1_ only (Figs 12 and 13). In cases of selective stimulation of the deeper nerve, both fibres are activated (an action potential is generated), but the shallower one (*N*_1_) is inhibited by a hyperpolarisation in the membrane potential, produced by either the negative current responsible for the excitation (Fig 15), or by positive current at the last electrode (Fig 14). Investigating these cases further, it could be observed that the selectivity is in general attributable to the effect of currents from the last two electrodes, which determine the nerve’s final state of excitation and/or inhibition. Excitations or inhibitions are the result of producing positive membrane potential in at least 10 consecutive nodes or a negative membrane potential in at least 15 consecutive nodes, respectively.

Results showed that it was indeed possible to selectively stimulate either fibre using fewer active electrodes. However, the number of cases of interest (selective activation of *N*_3_) dropped as the number of electrodes was reduced. Examination of the 8-electrode results (see examples above) suggests that selective activation of *N*_3_ is largely attributable to electrodes 7 and 8, or sometimes 6, 7 and 8. Therefore, reducing the stimuli to three electrodes disrupts some of these patterns, and reducing to two electrodes disrupts all of them, at least to some extent.

These results suggest that the simulation environment presented here could in future be used for optimisation of hardware design for selective stimulation. Although selective excitation is possible using only two or three electrodes, eight electrodes give greater flexibility in stimulus design, allowing a combination of localised activations or inhibitions at different positions in the fibre.

Summarising, the responses of the modelled fibres were consistent with preceding studies and experimental results [16, 39]. The selective stimulation results for the presented scenarios demonstrate the capabilities and extent of the simulation environment. In spite of the environment’s lack of detail in some aspects of the representation, it was able to emulate known responses for modelled nerve fibres, suggesting that it can meaningfully be used to derive new hypotheses for future testing in psychophysical studies.

## Conclusion

As demonstrated throughout this manuscript, the presented simulation environment provides an important tool for studying TENS in general and selective nerve stimulation in particular. It allows investigation of the design of electrode arrays for a TENS system in terms of electrode shape, spacing and number of electrodes, as well as studying the effect of different stimulation patterns. There is also a possibility of modelling nerves situated deeper than those considered in the present study; e.g., motor nerves. The presented model is a simplified representation of a human finger. Its level of complexity, however, was shown to be sufficient to produce simulation results that agree well with experimental results known from literature [16, 39].

The finger FEM developed for this work does not consider the capacitive and dielectric properties of the skin, fat, bone and the electrode system. Future versions of the simulation environment may include these aspects, which would improve the modelling of transients and other high-frequency effects. This would be useful for investigating the effect of frequency and width of the current pulses, as studied by Medina and Grill [44], but such an implementation would require data on the electrical properties of a real human finger that are at present unavailable. The environment could also be improved by including variable electrode shapes in the FEM, instead of having fixed rectangles as the current design, allowing electrode shape to be included when optimising the design of the array. Regarding the improvement of the nerve-fibre model, a non-linear double cable model [12] can be implemented to provide a more realistic nerve response. However, this would involve a higher computational cost because the number of sections in which the fibre is divided would be tripled (due to consideration of the paranodal and internodal sections of the fibre).

In addition to potential improvements of the simulation environment along the aforementioned lines, future work will be directed towards psychophysical experiments to support the simulation results, particularly in relation to the stimulation of nerve fibres located at different depths.

## Appendix—Set of equations for the nerve response model

This section contains all the equations needed for the computation of the nerve response model, starting from the electrical circuit representation and relating it to the HH equations.

The injected membrane current at the *n*th node *I*_*inj*,*n*_ is the sum of the currents flowing through the capacitor *C*_*m*_ and the membrane conductance *G*_*m*,*n*_ as follows:

$$\begin{array}{c} I_{inj,n}=C_{m}\frac{dV_{n}}{dt}+I_{i,n}, \end{array}$$ (1)

where *V*_*n*_ is the reduced membrane potential (see Fig 2) and the total ionic current is the sum of the sodium, potassium and leakage currents *I*_*i*,*n*_ = *I*_*Na*,*n*_ + *I*_*K*,*n*_ + *I*_*L*,*n*_. These ionic currents are described by the HH equations, which represent the dynamic behaviour (opening and closing) of the ion channels, controlled (see below) by the gating variables *n*, *m*, and *h* ∈ (0,1). This behaviour is determined by (2) to (4), in which the values of *α* and *β* are computed according to Eqs (5) to (7) using documented values for the human nerve fibre for the constants *A*, *B*, *C*, *D*, *Q*_10_ factor (see Table 5) and environmental temperature *T*, as follows:

$$\begin{array}{cc} {\dot{m}}_{n} & =[-\left(\alpha_{m}\left(V_{n}\right)+\beta_{m}\left(V_{n}\right)\right)m_{n}+\alpha_{m}\left(V_{n}\right)], \end{array}$$ (2)

$$\begin{array}{cc} {\dot{n}}_{n} & =[-\left(\alpha_{n}\left(V_{n}\right)+\beta_{n}\left(V_{n}\right)\right)n_{n}+\alpha_{n}\left(V_{n}\right)], \end{array}$$ (3)

$$\begin{array}{cc} {\dot{h}}_{n} & =[-\left(\alpha_{h}\left(V_{n}\right)+\beta_{h}\left(V_{n}\right)\right)h_{n}+\alpha_{h}\left(V_{n}\right)], \end{array}$$ (4)

$$\begin{array}{cc} \alpha_{m,n}\left(V_{n}\right) & =1000AQ_{10}^{(T-T_{0})/10}\frac{B-1000CV_{n}}{De^{B-1000CV_{n}}-1}, \end{array}$$ (5)

$$\begin{array}{cc} \beta_{m,n}\left(V_{n}\right),\alpha_{h}\left(V_{n}\right) & =1000AQ_{10}^{(T-T_{0})/10}e^{-\frac{1000V_{n}}{C}}, \end{array}$$ (6)

$$\begin{array}{cc} \beta_{h}\left(V_{n}\right) & =1000AQ_{10}^{(T-T_{0})/10}\frac{1}{e^{B-1000CV_{n}}+1}. \end{array}$$ (7)

The ion conductances and the maximum membrane conductances are then described by:

$$\begin{array}{cc} G_{Na_{n}} & =m_{n}^{3}h_{n}G_{Na,max}, \end{array}$$ (8)

$$\begin{array}{cc} G_{K_{n}} & =n_{n}^{4}G_{K,max}, \end{array}$$ (9)

where the conductances *G*_*ion*,*max*_ are calculated from known values *g*_*ion*_ of conductance per unit area of membrane (Table 4) using:

$$\begin{array}{c} G_{ion,max}=\pi dLg_{ion}\;\;\;\text{for}\;ion=Na,K,L. \end{array}$$ (10)

Referring to the electrical equivalent circuit (Fig 2), it can be seen that the ion currents are given by:

$$\begin{array}{cc} I_{Na,n} & =G_{Na,n}\left(V_{n}-V_{Na,max}\right), \end{array}$$ (11)

$$\begin{array}{cc} I_{K,n} & =G_{K,n}\left(V_{n}-V_{K,max}\right), \end{array}$$ (12)

$$\begin{array}{cc} I_{L,n} & =G_{L,n}\left(V_{n}-V_{L,max}\right). \end{array}$$ (13)

Thus, the HH model defines the total ionic current as:

$$\begin{array}{c} \begin{array}{c} I_{i,n}=G_{Na,max}m_{n}^{3}h_{n}\left(V_{n}-V_{Na,max}\right)+G_{K,max}n_{n}^{4}(V_{n}\\ -V_{K,max})+G_{L,max}\left(V_{n}-V_{L,max}\right). \end{array} \end{array}$$ (14)

In Eq (14), the channel reversal potentials *V*_*Na*,*max*_, *V*_*K*,*max*_ and *V*_*L*,*max*_ come from the Nernst equation [8]:

$$\begin{array}{c} V_{ion,max}=\frac{RT_{K}}{F}\ln(\frac{{\left[ion\right]}_{o}}{{\left[ion\right]}_{i}})-V_{r}\;\;\;\;\text{for}\;ion=Na,K,L, \end{array}$$ (15)

where *T*_*k*_ is the temperature in Kelvin, *R* the universal gas constant, *F* the Faraday constant and [*ion*]_*o*_/[*ion*]_*i*_ the extracellular to intracellular ion concentration ratio for sodium, potassium and leakage ions. The intracellular conductance *G*_*a*_ (see Fig 2) is calculated from the specific resistivity *ρ*_*i*_, as follows:

$$\begin{array}{c} G_{a}=\frac{\pi d^{2}}{4\rho_{i}\Delta x}. \end{array}$$ (16)

Considering all *N* nodes in the nerve fibre, the current flowing through each is described by:

$$\begin{array}{c} I_{inj,n}=\{\begin{array}{c} \left(V_{i,n+1}-V_{i,n}\right)G_{a}\\ \text{for}\;n=1,\\ \left(V_{i,n+1}-2V_{i,n}+V_{i,n-1}\right)G_{a}\\ \text{for}\;n\in (2,3,...,N-1),\\ \left(V_{i,n}+V_{i,n-1}\right)G_{a}\\ \text{for}\;n=N. \end{array} \end{array}$$ (17)

Combining (17) with (1) and (14), and using *V*_*i*,*n*_ = *V*_*n*_ + *V*_*e*,*n*_ + *V*_*r*_ (where *V*_*r*_ is the resting potential, see Fig 2) [7], the potential along the fibre (for all the *N* nodes) is described by the non-linear Eq (18), using the matrices (19), (20) and (21):

$$\begin{array}{c} V=\frac{1}{C_{m}}\int -I_{i}+G\left(V+V_{e}\right)dt, \end{array}$$ (18)

$$\begin{array}{c} V={\left[\begin{array}{c} V_{1}\\ \vdots \\ V_{N} \end{array}\right]}_{N\times 1}, \end{array}$$ (19)

$$\begin{array}{c} G={\left[\begin{array}{cccccc} -G_{a} & G_{a} & 0 & 0 & \cdots & 0\\ G_{a} & -2G_{a} & G_{a} & 0 & & 0\\ 0 & G_{a} & -2G_{a} & \ddots & \ddots & \vdots \\ 0 & 0 & \ddots & \ddots & G_{a} & 0\\ \vdots & & \ddots & G_{a} & -2G_{a} & G_{a}\\ 0 & \cdots & & 0 & G_{a} & -G_{a} \end{array}\right]}_{N\times N}, \end{array}$$ (20)

$$\begin{array}{c} I_{i}={\left[\begin{array}{c} \begin{array}{c} {}_{[-G_{Na},max}m_{1}^{3}h_{1}\left(V_{1}-V_{Na,max}\right)\\ -G_{K,max}n_{1}^{4}\left(V_{1}-V_{K,max}\right)-G_{L,max}\left(V_{1}-V_{L,max}\right)]\\ \vdots \\ {}_{[-G_{Na},max}m_{N}^{3}h_{N}\left(V_{N}-V_{Na,max}\right)\\ -G_{K,max}n_{N}^{4}\left(V_{N}-V_{K,max}\right)-G_{L,max}\left(V_{N}-V_{L,max}\right)] \end{array} \end{array}\right]}_{N\times 1}, \end{array}$$ (21)

Likewise, the gating variables are given by (22), (23) and (24):

$$\begin{array}{c} \begin{array}{cc} \dot{m} & ={\left[\begin{array}{c} {\dot{m}}_{1}\\ \vdots \\ {\dot{m}}_{N} \end{array}\right]}_{N\times 1}\\ & ={\left[\begin{array}{c} \left(-\left(\alpha_{m}\left(V_{1}\right)+\beta_{m}\left(V_{1}\right)\right)m_{1}+\alpha_{m}\left(V_{1}\right)\right)\\ \vdots \\ \left(-\left(\alpha_{m}\left(V_{N}\right)+\beta_{m}\left(V_{N}\right)\right)m_{N}+\alpha_{m}\left(V_{N}\right)\right) \end{array}\right]}_{N\times 1}, \end{array} \end{array}$$ (22)

$$\begin{array}{c} \begin{array}{cc} \dot{n} & ={\left[\begin{array}{c} {\dot{n}}_{1}\\ \vdots \\ {\dot{n}}_{N} \end{array}\right]}_{N\times 1}\\ & ={\left[\begin{array}{c} \left(-\left(\alpha_{n}\left(V_{1}\right)+\beta_{n}\left(V_{1}\right)\right)n_{1}+\alpha_{n}\left(V_{1}\right)\right)\\ \vdots \\ \left(-\left(\alpha_{n}\left(V_{N}\right)+\beta_{n}\left(V_{N}\right)\right)n_{N}+\alpha_{n}\left(V_{N}\right)\right) \end{array}\right]}_{N\times 1}, \end{array} \end{array}$$ (23)

$$\begin{array}{c} \begin{array}{cc} \dot{h} & ={\left[\begin{array}{c} {\dot{h}}_{1}\\ \vdots \\ {\dot{h}}_{N} \end{array}\right]}_{N\times 1}\\ & ={\left[\begin{array}{c} \left(-\left(\alpha_{h}\left(V_{1}\right)+\beta_{h}\left(V_{1}\right)\right)h_{1}+\alpha_{h}\left(V_{1}\right)\right)\\ \vdots \\ \left(-\left(\alpha_{h}\left(V_{N}\right)+\beta_{h}\left(V_{N}\right)\right)h_{N}+\alpha_{h}\left(V_{N}\right)\right) \end{array}\right]}_{N\times 1}. \end{array} \end{array}$$ (24)

The constants *C*_*m*_, *G*_*a*_, *V*_*Na*,*max*_, *V*_*K*,*max*_, *V*_*L*,*max*_, *G*_*Na*,*n*_, *G*_*K*,*n*_ and *G*_*L*,*n*_ directly depend on the node size.
